# AlN Nanowall Structures Grown on Si (111) Substrate by Molecular Beam Epitaxy

**DOI:** 10.1186/s11671-015-1178-7

**Published:** 2015-12-01

**Authors:** Yosuke Tamura, Kazuhiro Hane

**Affiliations:** Department of Nanomechanics, Graduate School of Engineering Tohoku University, 6-6-01, Aramaki Aza Aoba, Aoba-ku, Sendai, Japan

**Keywords:** AlN, Nanostructure, Nanowall, MBE, Si substrate, Epitaxial growth, XRD, 81.07.BC, 61.46.Hk, 61.46.Df

## Abstract

AlN nanowall structures were grown on Si (111) substrate using molecular beam epitaxy at substrate temperature of 700 °C with N/Al flux ratios ranging from 50 to 660. A few types of other AlN nanostructures were also grown under the nitrogen-rich conditions. The AlN nanowalls were ranged typically 60–120 nm in width and from 190 to 470 nm in length by changing N/Al flux ratio. The AlN nanowall structures grown along the c-plane consisted of AlN (0002) crystal with full-width at half maximum of the rocking curve about 5000 arcsec.

## Background

For the last few decades, III-nitride compounds consisting of GaN, InN, and AlN crystals attract strong interests in optical and electronic research fields due to the superior characteristics such as widely tunable bandgap [1–6]. Especially, AlN crystals are promising for deep-ultraviolet light-emitting diodes and laser diodes because of the wide direct-transition bandgap (6.2 eV) [7, 8]. Nanostructures of III-nitride compounds are also studied intensively. Nano-rods/pillars/wires are widely investigated [2–6]. Comparing with bulk crystals, new properties caused by the nanoscale sizes are expected. In addition, the hetero-epitaxial growth of the nanostructures on Si substrate is often easier than the bulk crystal growth on Si substrate, which is useful for monolithic electronic circuit integration.

In the case of GaN nanostructures, GaN nanopillars were studied intensively. Several excellent properties, such as lasing [9], high-efficient emission at a high-indium concentration [10], and periodically arranged pillars [11] were reported. However, a disadvantage of nanopillar structures is the electrical disconnection to individual nanopillars. The grown nanopillars are usually isolated from each other although several connection techniques are studied [12]. Nanopillars of AlN crystals were also investigated. However, the reports were few [7, 13, 14].

As a different morphology, GaN nanowall structures were reported recently under nitrogen-rich growth condition of molecular beam epitaxy (MBE). The GaN nanowalls were grown on sapphire substrate [4, 15]. The GaN nanowalls were also grown on Si (111) substrates [16, 17]. The nanowalls were usually connected to construct a honeycomb-like network structure. And thus, the electrical current could flow along the in-plane direction. The GaN nanowall had Ga-polarity, and the width of GaN nanowall was controlled by varying N/Ga flux ratio. Depositing a platinum metal electrode as a Schottky contact on the GaN nanowall network, a Schottky diode hydrogen sensor was demonstrated [18, 19].

On the other hand, there are very few reports on AlN nanowall structure. Since GaN and AlN crystals are similar in crystalline structure, it is worthwhile to investigate whether nanowall structure can be grown in the case of AlN crystal growth. In this paper, AlN nanowall structures are grown on Si (111) substrates by MBE. Relationship between N/Al flux ratio and nanowall structure is investigated. The crystal qualities of the nanowalls are also studied.

## Methods

The AlN crystals were grown on a Si (111) substrate (thickness 380 μm, resistance ≤0.02 Ω m) using MBE system (RIBER 32; RIBER) with radio frequency (RF) plasma source (RFS-N/TH; Veeco Instruments). As the nitrogen and aluminum sources, we used nitrogen gas with the purity of 99.99995 % and solid metal aluminum with the purity of 99.9999 %.

First, Si (111) substrate was cleaned in the standard RCA cleaning process, then the native oxide of the Si substrate surface was removed and the Si surface was terminated with hydrogen by a diluted hydrogen fluoride solution (HF; H_2_O = 1:100). After substrate was dried by nitrogen blowing, the substrate was transferred in a vacuum chamber and hydrocarbons of Si substrate surface were removed by pre-heating around 10^−5^ Pa [20]. After the Si substrate was transferred to the growth chamber, several monolayers of aluminum were deposited to avoid nitridation of Si surface. Finally, nitrogen plasma was ignited and AlN nanowall structures were grown on the Si substrates at 700 °C. The RF plasma source power was fixed at 400 W. AlN crystals were grown in the N/Al flux ratios of 50, 200, 400, 550, and 660. The N/Al flux ratio was determined by the ratios of nitrogen flux and aluminum flux, which were beam equivalent pressures measured by Bayard-Alpert gauge. In order to vary the N/Al flux ratio, the aluminum flux was kept constant at 7.6 × 10^−8^ Torr and the nitrogen flux was changed.

The crystal morphology of the grown AlN crystal was evaluated by a field-emission scanning electron microscopy (SU-70, Hitachi) and the crystal structure was measured by an X-ray diffraction machine (XRD; D8 Discover, Bruker).

## Results and Discussion

**Fig. 1:**
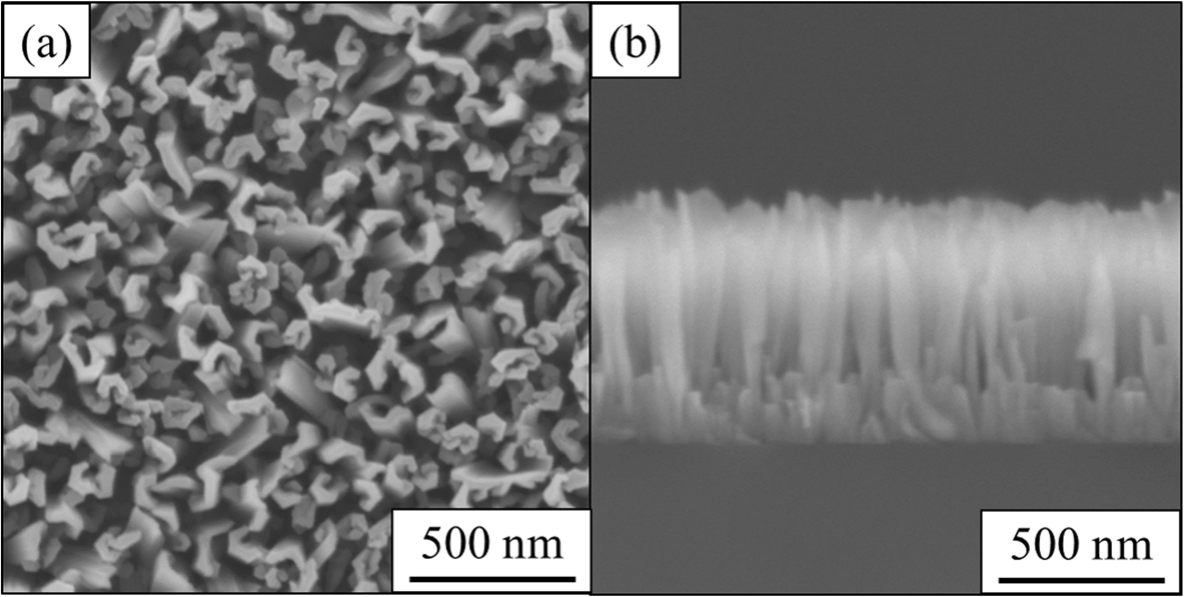
Scanning electron micrographs of AlN nanowall structure grown on Si (111) substrate. **a** Top view. **b** Cross-sectional view

Figure 1a, b shows a top view and a cross-sectional view of scanning electron micrograph of an AlN crystal structure grown on Si (111) substrate in the N/Al flux ratio of 200. AlN nanowall structures are seen. From Fig. 1a, the width of the nanowall is from 40 to 80 nm on the top of the nanowalls. The nanowall extends typically 470 nm in horizontal length. The typical length is obtained from the top surface area of the AlN nanowalls divided by the nanowall average width and the density of AlN nanowalls. As shown in Fig. 1b, the thickness of the AlN nanowall structure is 750 nm. The nanowall consists of several pillars connected in the upper parts. The pillars start to grow from the Si substrate and become wider when growing. Comparing with GaN nanowall structures, the horizontal extension of AlN nanowalls are limited and they do not form a fully connected network structure [4, 15–19].

**Fig. 2:**
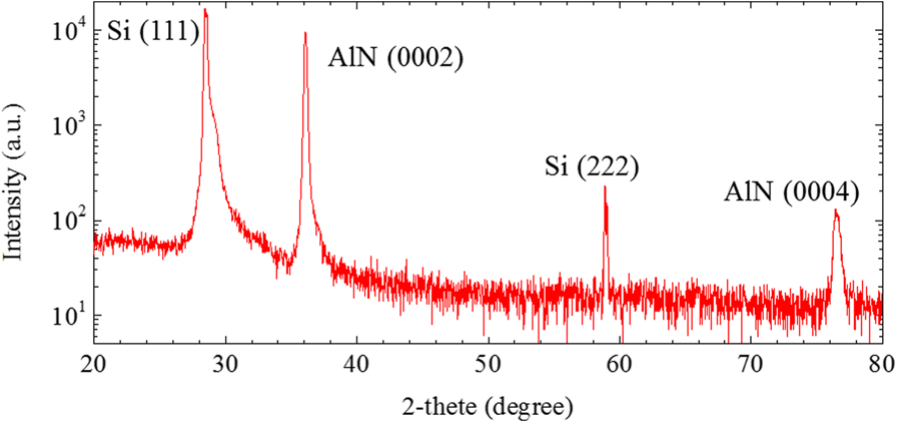
XRD pattern of AlN nanowall structure grown on Si (111) substrate in N/Al flux ratio of 200

To investigate the crystal structure of the AlN nanowall, XRD measurement of 2*θ*-ω scan was carried out. The diffraction pattern is shown in Fig. 2. As shown in the figure, four strong peaks are seen. According to the database, these peaks at 28.5°, 36.1°, 58.8°, and 76.5° are corresponding to the crystalline signals of Si (111), AlN (0002), Si (222), and AlN (0004), respectively. Therefore, the grown AlN nanowall consists of hexagonal AlN crystal along c-axis.

**Fig. 3:**
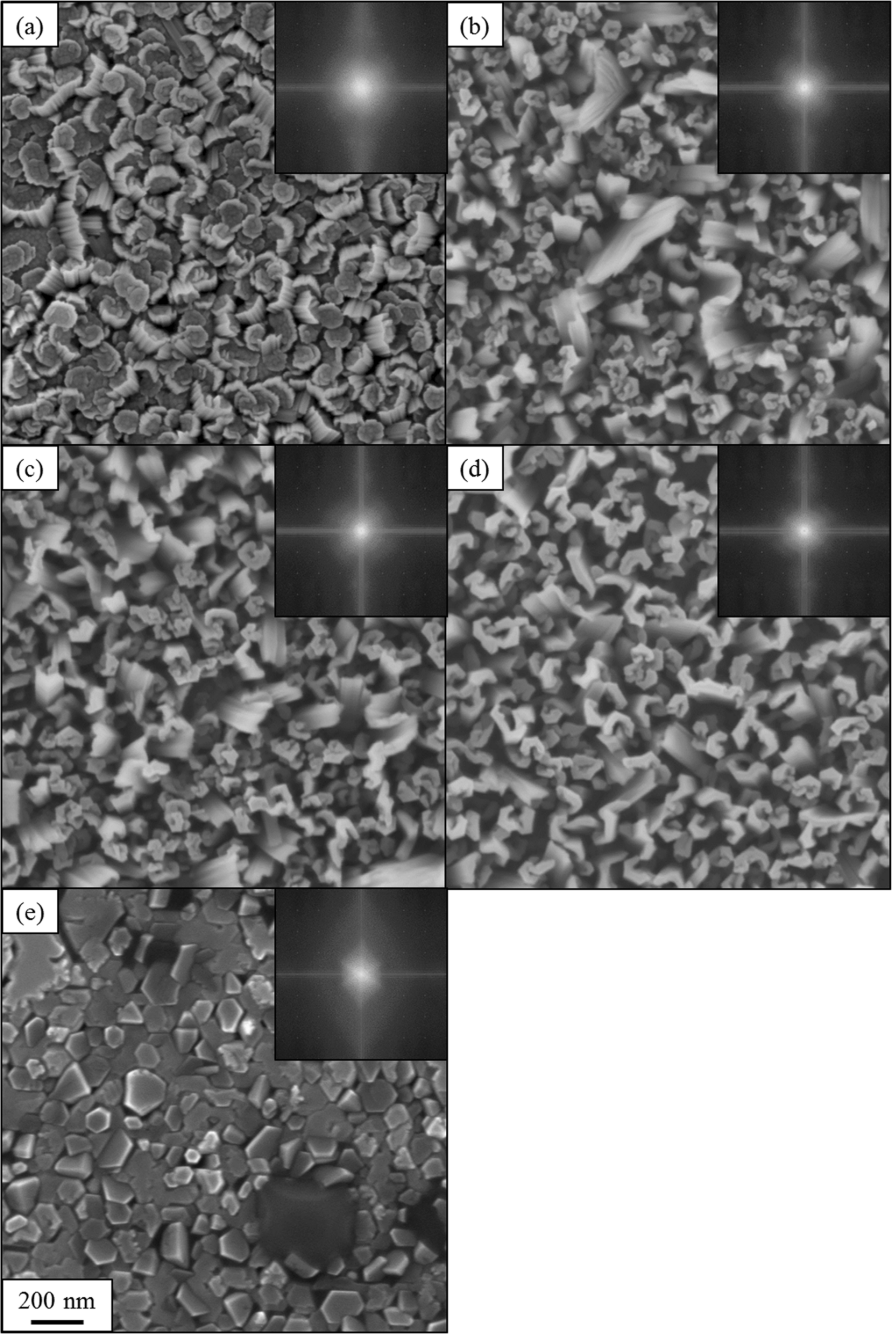
Scanning electron micrographs of AlN nanowall structures grown on Si (111) substrate in the different N/Al flux ratios **a** 660, **b** 550, **c** 400, **d** 200, and **e** 50. The inserts are the fast Fourier transformed patterns

The AlN crystal was grown in the different N/Al flux ratio at the substrate temperature of 700 °C. The aluminum flux was kept constant at the pressure of 7.6 × 10^−8^ Torr and the nitrogen flux was changed. Figure 3a–e shows top views of scanning electron micrographs of the AlN nanowall structures grown in the N/Al flux ratios ranging from 50 to 660. The inserts show the fast Fourier transform (FFT) patterns of the respective images.

In the case of the high N/Al flux ratio of 660 shown in Fig. 3a, the dense short AlN nanocolumns are seen. In the large nitrogen flux condition, the aluminum adatoms easily collide with nitrogen adatoms. Therefore, the 3-dimensional nucleation is dominant [21–23]. Moreover, the blurred FFT pattern is observed, which indicates somewhat a random formation of AlN nanostructures. In the N/Al flux ratio between 200 and 550, the AlN nanowall structures are observed as shown in Fig. 3b–d. In this range, with the increasing N/Al flux ratio, the average width of AlN nanowall structures increases typically from 60 to 120 nm. The horizontal length of AlN nanowall decreases typically from 470 to 190 nm. The FFT patterns do not show clear symmetry. On the other hand, in the low N/Al flux ratio of 50 shown in Fig. 3e, the dense hexagonal AlN structures are observed. Generally, to grow AlN films, the atom fluxes of nitrogen and aluminum are equal. This condition is obtained by decreasing N/Al flux ratio in our experiment. The flat AlN crystal planes appeared as shown in Fig. 3e. In this condition, sixfold symmetry FFT pattern is obtained as seen in the inset of Fig. 3e.

**Fig. 4:**
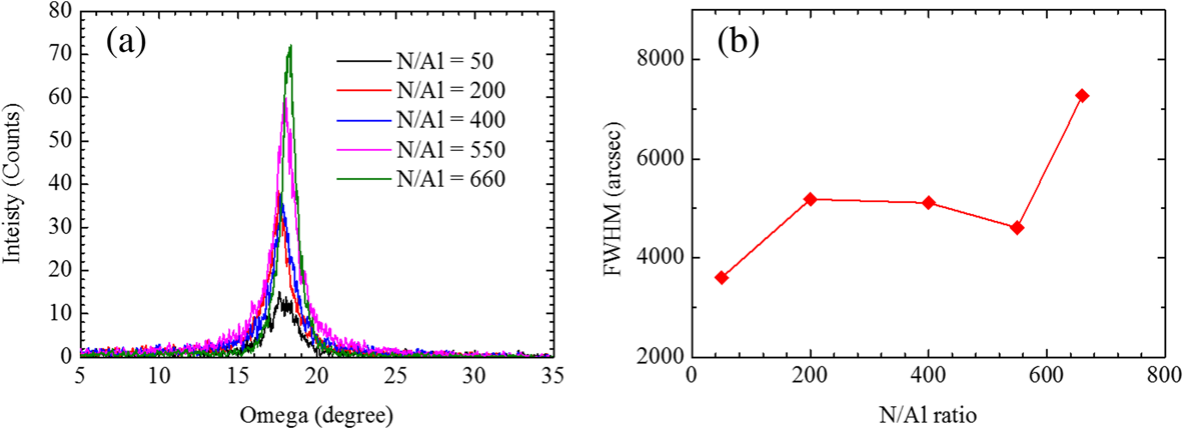
**a** Rocking curve spectra of AlN (0002). **b** Rocking-curve width of AlN (0002) as a function of N/Al flux ratio

To investigate crystal quality of the AlN nanostructures, XRD rocking curve was measured in each N/Al flux ratio. Figure 4a shows the rocking curve spectra of AlN (0002). From these spectra, the values of full width at half maximum (FWHM) were measured as shown in Fig. 4b. The value of FWHM is approximately 3600 arcsec in the N/Al flux ratio of 50, which is the narrowest value in this experiment. From the ratio from 200 to 550, the value of FWHM is around 5000 arcsec. In the ratio of 660, the value of FWHM increases to 7300 arcsec.

**Fig. 5:**
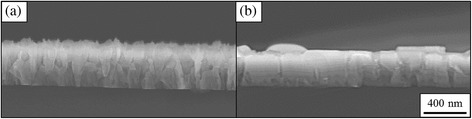
Scanning electron micrographs of AlN nanowall structures grown on Si (111) substrate at the N/Al flux ratios **a** 660 and **b** 50

Figure 5a, b shows the cross-sectional images of AlN nanostructures which are grown in the N/Al flux ratios of 660 and 50. In the case of the N/Al flux ratio of 660 as shown in Fig. 5a, many grain boundary of the AlN nanostructures are seen which extend from the interface of Si substrate to the top surface of AlN layers. Considering the broaden FWHM value, the AlN nanostructures have many different and irregular crystal orientations.

On the other hand, in the case of the low N/Al flux ratio of 50 (Fig. 5b), the wider AlN nanostructures are seen, which consist of the crystals extending continuously from the bottom to the top of the AlN layer. This fact and the narrow FWHM suggest that the crystal quality of the AlN nanostructures is better than the others. The AlN nanowall structures appear between these conditions, the N/Al flux ratio from 200 to 550.

AlN crystalline films were often grown by metalorganic chemical vapor deposition. The better crystal quality was reported because the AlN crystals were grown at the higher temperature [24]. In the case of MBE, the crystal quality was improved using a high substrate temperature around 800 °C and inserting buffer layers [25, 26]. Unlike these, the AlN nanowall structures appeared at the substrate temperature of 700 °C in the high N/Al flux ratio at the expense of the crystal quality.

## Conclusions

The AlN nanowall structures were directly grown on Si (111) substrate at the substrate temperature at 700 °C by using RF-MBE without any templates or catalysis. From XRD spectra, the AlN nanowall structures consist of the hexagonal AlN crystals grown along c-plane. The AlN nanowall structures were grown in the N/Al flux ratio from 200 to 550. The width of AlN nanowall was varied from 60 to 120 nm increasing N/Al flux ratio and the length were from 470 to 190 nm. The AlN nanowalls were grown in the nitrogen-rich growth condition at the expense of the crystal quality. The AlN nanowall structures can be valuable for adsorption devices using the large surface volume ratio such as demonstrated for GaN nanowall network crystals [19, 20].
